# Integrated multi-omics identification of m6A-SNP-related diagnostic biomarkers in amyotrophic lateral sclerosis

**DOI:** 10.3389/fimmu.2026.1914869

**Published:** 2026-08-27

**Authors:** Hongfen Wang, Bo Sun, Shiya Wang, Jing Pan, Rongrong Du, Xinyuan Pang, Jiongming Bai, Jinming Han, Jinli Feng

**Affiliations:** 1 Senior Department of Neurology, Chinese People's Liberation Army General Hospital, Beijing, China; 2 Department of Neurology, Beijing Chaoyang Hospital, Capital Medical University, Beijing, China; 3 Outpatient Department of Zhantansi, Jingzhong Medical Zone, Chinese People's Liberation Army General Hospital, Beijing, China; 4 Department of Respiratory and Critical Care Medicine, Second Medical Center, Chinese People's Liberation Army General Hospital, Beijing, China; 5 Department of Neurology, Xuanwu Hospital, Beijing, China

**Keywords:** amyotrophic lateral sclerosis, biomarkers, m6A-SNP, multi-omics, N6-methyladenosine

## Abstract

**Background:**

Amyotrophic lateral sclerosis (ALS) lacks reliable and minimally invasive biomarkers for early diagnosis. m6A-associated single-nucleotide polymorphisms (m6A-SNPs) may influence RNA methylation and gene expression, offering opportunities to identify clinically relevant diagnostic markers.

**Methods:**

We integrated eQTLGen cis-eQTL data, RMVar m6A-SNP annotations, and ALS transcriptomic datasets to identify m6A-SNP–related genes. Random Forest and LASSO regression were combined to screen robust diagnostic markers. A nomogram was constructed and validated using independent cohorts. Immune infiltration, predicted m6A modification sites, and potential RBP–SNP interactions were assessed. Peripheral blood samples from ALS patients were used for exploratory validation of gene expression and global m6A levels.

**Results:**

We identified 109 ALS-associated m6A-SNP-related genes with cis-eQTL signals and narrowed these to seven candidate diagnostic markers (TMED5, OXR1, BRI3, FEM1C, SUZ12, EIF2AK4, and TJAP1). The seven-gene model outperformed the individual markers in the training cohort and retained moderate discrimination in the independent validation cohort. ALS samples showed differences in inferred immune-cell composition, including monocytes, neutrophils, and T-cell subsets. The selected SNP loci were located near predicted m6A sites and annotated RBP-binding regions. Exploratory clinical validation showed significant upregulation of FEM1C and SUZ12 at both mRNA and protein levels, accompanied by reduced global m6A modification.

**Conclusions:**

Through multi-omics integration and exploratory clinical validation, this study identifies m6A-SNP-related candidate markers associated with ALS. The findings support further evaluation of m6A-related signatures for ALS discrimination and molecular characterization, while larger independent cohorts and additional calibration are required before clinical application.

## 1 Background

Amyotrophic lateral sclerosis (ALS) is a fatal neurodegenerative disorder characterized by progressive degeneration of upper and lower motor neurons, resulting in muscle weakness, atrophy, and ultimately respiratory failure (1). Despite advances in understanding ALS genetics, early diagnosis remains challenging, and effective treatments remain limited (2–4).

The etiology of ALS remains largely unknown, with approximately 10% of cases linked to specific genetic mutations (familial ALS). The remaining 90% of cases manifest as sporadic ALS (5). This type is associated with environmental factors, including exposure to toxins and infections (6, 7). Although the majority of ALS cases are sporadic, genetic studies have revealed substantial molecular heterogeneity underlying ALS pathogenesis (7). More than 30 genes have been implicated in ALS susceptibility, with several major causative genes accounting for a significant proportion of familial and sporadic cases (8). Among these, C9orf72 repeat expansion, SOD1, TARDBP, and FUS are recognized as the most prevalent genetic contributors to ALS. The hexanucleotide repeat expansion in C9orf72 represents the most common genetic cause of ALS and frontotemporal dementia (FTD), accounting for a considerable proportion of familial ALS cases in European populations (9). Mutations in SOD1 cause ALS through mechanisms involving oxidative stress, mitochondrial dysfunction, and impaired protein homeostasis, whereas mutations in RNA-binding proteins such as TARDBP and FUS highlight the critical role of RNA metabolism dysregulation in ALS pathogenesis (10–12). In addition, other genes including TBK1, OPTN, VCP, UBQLN2, NEK1, and KIF5A have been identified as ALS-associated genes, further demonstrating the complex genetic architecture of this disease (13, 14).

Importantly, ALS and FTD are increasingly recognized as overlapping manifestations of a common neurodegenerative disease spectrum rather than completely distinct disorders. Approximately 10-15% of ALS patients develop clinical features of FTD, while a larger proportion exhibit subclinical cognitive and behavioral impairments associated with frontotemporal dysfunction (15, 16). Conversely, a subset of FTD patients develop motor neuron degeneration resembling ALS, further supporting the existence of an ALS–FTD disease continuum (17). The pathological link between ALS and FTD is primarily characterized by abnormal aggregation and mislocalization of the RNA-binding protein TDP-43, which is observed in the majority of ALS cases and approximately half of FTD cases (18, 19). Given that several ALS/FTD-associated genes, including C9orf72, TARDBP, FUS, and TBK1, are directly involved in RNA processing, RNA transport, and cellular homeostasis (20, 21), dysregulation of post-transcriptional regulatory mechanisms may represent a convergent molecular mechanism underlying ALS-FTD spectrum disorders (1).

Epigenetic regulation, including DNA methylation, histone modifications, and mRNA methylation, plays a growing role in the study of ALS pathogenesis (22). Among epitranscriptomic modifications, N6-methyladenosine (m6A) is a prevalent post-transcriptional modification in eukaryotic mRNA and influences mRNA stability, splicing, and translation (23–25). Increasing evidence suggests that m6A dysregulation is involved in neurodegenerative diseases, including Alzheimer’s disease (AD) and Parkinson’s disease (PD) (25, 26), and may contribute to RNA- and protein-homeostasis pathways relevant to TDP-43 pathology.

Thus, investigation of m6A modifications in ALS may support biomarker discovery and mechanistic studies. Previous integrated genomic analyses have identified m6A-SNP risk loci in other neurodegenerative diseases, including AD and PD (27–29). However, ALS-associated m6A-SNPs have not been systematically investigated, and their diagnostic relevance remains uncertain.

## 2 Methods

### 2.1 Experimental workflow of the ALS m6A-SNP integrated analysis study

### 2.2 Clinical sample collection

The peripheral blood validation cohort included 10 patients with definite ALS and 10 healthy controls. Patient-level clinical characteristics of the ALS group are presented in **Table 1**, while age and sex comparisons between ALS patients and controls are summarized in **Table 2**. Patients were eligible for inclusion if they met the revised El Escorial criteria for definite ALS (30). This exploratory clinical validation study was approved by the Ethics Committee of the Chinese PLA General Hospital, Beijing, China (approval no. S2024-405-01), and informed consent was obtained from all participants or their legal guardians.

**Table 1:** Clinical characteristics of ALS patients (n=10).

Patient ID	Age	Sex	Site of onset	Disease duration (months)	ALSFRS-R	Family history	Mutation status	Exclusion of other causes
Patient 1	52	Male	Upper limb	36	37	Negative	Negative	Yes
Patient 2	47	Male	Lower limb	12	42	Negative	Negative	Yes
Patient 3	68	Male	Upper limb	11	43	Negative	Negative	Yes
Patient 4	60	Male	Upper limb	24	41	Negative	Negative	Yes
Patient 5	44	Female	Upper limb	8	41	Negative	Negative	Yes
Patient 6	63	Male	Bulbar	12	45	Negative	Negative	Yes
Patient 7	61	Female	Bulbar	12	41	Negative	Negative	Yes
Patient 8	53	Female	Lower limb	12	40	Negative	Negative	Yes
Patient 9	54	Male	Upper limb	24	45	Negative	Negative	Yes
Patient 10	53	Male	Upper limb	7	47	Negative	Negative	Yes

**Table 2:** Baseline demographic comparison of ALS patients (n=10) and healthy controls (n=10).

Variable	ALS patients	Healthy control	*P* value
Age (years), mean ± SD	55.5 ± 7.41	48.8 ± 14.76	0.216
Male sex, n (%)	7 (70.0%)	6 (60.0%)	0.639

### 2.3 Data retrieval

Blood expression quantitative trait locus (eQTL) data were retrieved from the eQTLGen consortium (31). This resource reports large-scale cis- and trans-eQTL analyses in blood, including data from 31,684 individuals. We used the available significant cis-eQTL results and allele-frequency information for downstream integration.

Datasets GSE112676 and GSE112680 were selected as our bulk analysis cohorts, with GSE112676 serving as a training set and GSE112680 as a validation set. We downloaded expression profile data and clinical data from the Gene Expression Omnibus (GEO) database.

GSE112676 included 741 whole-blood samples, including 233 ALS patients and 508 non-neurological disease controls. GSE112680 initially included 376 samples, including 164 ALS patients, 137 controls, and 75 ALS mimic cases. The ALS mimic samples were excluded before downstream analyses, resulting in 301 samples (164 ALS patients and 137 controls) in the final validation cohort. Both datasets were generated from PAXgene-stabilized whole blood samples. GSE112676 was based on the Illumina HumanHT-12 v3 Expression BeadChip platform (GPL6947), whereas GSE112680 was based on the Illumina HumanHT-12 v4 platform (GPL10558). The detailed characteristics of the GEO cohorts, including sample size, sample source, expression platform, and available clinical information, are summarized in **Supplementary Table 1**.

The GEO series matrix files had already undergone platform-specific preprocessing by the original data depositors, including normalization, log2 transformation, and quality-control procedures. The deposited metadata also documented technical procedures, including chip randomization and surrogate variable analysis (SVA), performed by the original investigators. These original preprocessing procedures were considered part of the GEO data processing and were distinguished from the downstream analyses performed in the present study.

For downstream processing, empty probes and probes mapping to multiple genes were removed. When multiple probes corresponded to the same gene, the median expression value was used as the final gene-level expression value.

The two GEO datasets were analyzed separately, with GSE112676 used as the training cohort and GSE112680 used as an independent external validation cohort. The datasets were not pooled for model construction, and batch-effect correction across datasets was therefore not performed in the present study. Available demographic and clinical characteristics, including sex distribution, age at disease onset, disease duration, and onset subtype, were summarized. Differences in sex distribution between ALS and control groups were evaluated using chi-square tests. Because age at onset, disease duration, and onset subtype were only available for ALS patients, direct age matching or adjustment across ALS and control groups could not be performed. Clinical variables including treatment information, disease duration at sampling, and blood-cell counts were unavailable in the GEO datasets and therefore were not included as covariates. Because direct blood-cell counts were unavailable, immune cell proportions were inferred from transcriptomic profiles using the CIBERSORT algorithm with the LM22 signature matrix. Correlations between inferred immune cell proportions and candidate marker genes were subsequently evaluated.

### 2.4 Analysis of ALS-associated N6-methyladenosine single nucleotide polymorphisms

The RNA Modification-associated Variants (RMVar) database (32) was used as the source of RNA modification-associated variants, including m6A-related SNP annotations. RMVar integrates genetic variants with multiple RNA modification types and provides variant-level information for exploring potential effects on RNA modification.

The RMVar database was used to cross-reference m6A-SNP data with eQTL data based on SNPs and genes. The resulting intersecting genes were further cross-checked against differentially expressed genes (DEGs) from our ALS bulk data to explore ALS-associated m6A-SNPs.

Genes harboring both m6A-SNP annotations and cis-eQTL signals were considered m6A-SNP-related genes. These genes were subsequently intersected with ALS-related differentially expressed genes to identify candidate m6A-SNP-associated markers.

### 2.5 Gene ontology enrichment analysis

To investigate the biological significance of the target genes, we performed functional enrichment analysis using the Metascape online platform. Gene ontology (GO) enrichment analysis describes the roles of genes in biological systems, including related biological processes (BPs), molecular functions (MFs), and cellular components (CCs). ALS-associated m6A-SNPs with a Benjamini-Hochberg (BH)-adjusted *p*-value <0.05 were considered significantly enriched.

### 2.6 Differential expression analysis

Differential expression analysis was performed on the preprocessed gene-level expression matrices using the limma R/Bioconductor package. Linear models were fitted with the lmFit function and moderated statistics were obtained with eBayes. Genes with |log2FC| >0.25 and an adjusted p-value <0.05 were considered differentially expressed. The results were visualized as heatmaps using the R package tidyheatmaps.

### 2.7 Machine learning screening for diagnostic marker genes

To identify candidate diagnostic marker genes for ALS, we applied an integrated machine-learning strategy combining random forest and LASSO logistic regression. The outcome variable was binary coded as ALS = 1 and control = 0. Candidate genes were obtained by intersecting genes containing m6A-SNPs with cis-eQTL signals and ALS-associated differentially expressed genes. A total of 109 candidate genes were used for machine-learning screening.

Key genes were screened using the random forest algorithm from the R *randomForest* package and the least absolute shrinkage and selection operator (LASSO) algorithm from the *glmnet* package. For random forest analysis, the mtry parameter was optimized using five-fold cross-validation, and a random forest model was constructed with 5,000 trees (ntree = 5000). Feature importance was evaluated according to MeanDecreaseGini values. The top 10 genes ranked by MeanDecreaseGini were selected as random forest-derived features.

For LASSO analysis, penalized logistic regression was performed using the R glmnet package with family = “binomial” and alpha = 1. The optimal lambda value was selected using ten-fold cross-validation based on minimum binomial deviance. Genes with non-zero coefficients at the optimal lambda (lambda.min) were retained as LASSO-selected features. Genes identified by both random forest and LASSO were considered candidate marker genes, and their stability was further evaluated by bootstrap resampling.

Feature stability was further evaluated using 100 stratified bootstrap resampling iterations. In each bootstrap iteration, ALS patients and controls were resampled while maintaining the original class proportion, and the complete feature-selection procedure, including random forest tuning, LASSO modeling, and feature intersection, was repeated independently. The selection frequency of each gene across the 100 iterations was calculated as the proportion of iterations in which the gene was selected. Genes with selection frequencies ≥req were considered stable features.

Cross-validation was used for hyperparameter tuning within the training cohort: five-fold cross-validation was used to optimize the random-forest mtry parameter and ten-fold cross-validation was used to select the LASSO penalty parameter. The final feature-selection pipeline was developed using GSE112676, whereas GSE112680 was kept separate and was used only for external validation. Because a complete nested cross-validation estimate of the entire feature-selection pipeline was not generated, training-cohort performance is reported as apparent performance and the unchanged-coefficient external validation is treated as the primary assessment of generalizability.

The seven-gene diagnostic model and nomogram were constructed in the training cohort using regression coefficients estimated from GSE112676. The derived coefficients were then applied unchanged to GSE112680 without coefficient re-estimation or model reconstruction. Receiver operating characteristic (ROC) curves were used to evaluate discrimination, with AUC values and 95% confidence intervals estimated using the DeLong method. Model calibration was assessed using the calibration intercept, calibration slope, Brier score, and Hosmer-Lemeshow goodness-of-fit test. Marker-specific and combined-model AUCs are summarized in **Supplementary Table 2**, and calibration results are reported in **Supplementary Table 3**. Recalibrated analyses in **Supplementary Table 3** are presented as sensitivity analyses and do not replace the primary unchanged-coefficient external validation.

### 2.8 Immune microenvironment analysis

The relative proportions of 22 immune cell types in the samples were estimated using the Type Identification by Relative Subset Estimation of RNA Transcripts (CIBERSORT) algorithm, implemented in the *IOBR* package. To assess whether immune cell infiltration profiles could distinguish patients with ALS from controls, principal component analysis (PCA) was performed using the *FactoMineR* package. Correlations between immune cell types were assessed with Spearman’s rank correlation in the *psych* package. A *p*-value of <0.05 was considered statistically significant.

CIBERSORT results were visualized using the *ggplot2* package, correlation heatmaps were generated with the *corrplot* package, and PCA results were visualized using the *factoextra* package.

### 2.9 Prediction of m6A modification sites

We used the sequence-based RNA adenosine methylation site predictor (SRAMP) (33) to identify predicted m6A modification sites near the m6A-SNPs corresponding to the genes selected by machine learning. SRAMP is a sequence-based prediction tool for mammalian m6A sites; accordingly, these analyses were treated as in silico predictions rather than direct experimental evidence of methylation.

### 2.10 Interactions between m6A-single nucleotide polymorphisms and RNA-binding proteins

To examine the genomic context of the selected m6A-SNPs and potential RNA-binding protein (RBP) regulatory regions, we used the UCSC Genome Browser (GRCh37/hg19) (34). Genome-browser annotations were used for hypothesis-generating assessment of whether the selected loci were located near annotated regulatory and RBP-binding regions.

### 2.11 Clinical sample collection and quantitative real-time polymerase chain reactions

3 mL peripheral blood samples were collected from patients diagnosed with ALS and controls using metal-free vacuum EDTA tubes (Becton Dickinson). Blood samples were stored at room temperature and analyzed within two hours of collection. We extracted total RNA from the human peripheral blood samples using TRIzol LS Reagent (Invitrogen, ThermoFisher) according to the manufacturer’s instructions. We used a NanoDrop spectrophotometer to measure RNA concentration and purity. Subsequently, 1 μg of RNA was reverse-transcribed into cDNA using a PrimeScript RT Reagent Kit (Takara Bio Inc., Kusatsu, Shiga, Japan). Finally, quantitative polymerase chain reactions (qPCR) were performed using SYBR Green qPCR reagents on a quantitative real-time PCR (qRT-PCR) instrument (ABI 7500), with glyceraldehyde-3-phosphate dehydrogenase (GAPDH) as the internal reference gene. Each sample was run in triplicate. The primers used are listed in **Table 3**.

**Table 3:** Primer sequences used for quantitative real-time polymerase chain reactions.

Gene	Primer sequence (5′-3′)
*TMED5*	*F: CTCGATAGCGACTTCACCTTTAC*
	*R: GGTGCTGAATGTATTGTCAAAGC*
*OXR1*	*F: TTCGACCAAACCTAAGTGATCCC*
	*R: GGGGTGTCTAAACCTGTCATTG*
*BRI3*	*F: TACCTCGTCACAGGGATACCC*
	*R: CGATAGAGTTGGCAGGATAGCG*
*FEM1C*	*F: GTGCCTCCATAGAAGTTGGGG*
	*R: CAGAAGCGGCCCATAAAGGG*
*SUZ12*	*F: AGGCTGACCACGAGCTTTTC*
	*R: GGTGCTATGAGATTCCGAGTTC*
*EIF2AK4*	*F: AAATGCCCACCTACCTATCCA*
	*R: CCTCCCCACAGTGTTTCTTGG*
*TJAP1*	*F: TGCAACAAGTCCCACTTCCG*
	*R: ATGTTTCCGAACCATGTCCTG*
*GAPDH*	*F: ACAACTTTGGTATCGTGGAAGG*
	*R: GCCATCACGCCACAGTTTC*

### 2.12 Western blot analysis

For every 1 mL of fresh anti-coagulated blood, 10 mL of red blood cell lysis buffer was added. The mixture was then incubated for 10 min. After lysis, the samples were centrifuged at 500 g for 5 min. The cell pellet was retained, and 300 μL of radioimmunoprecipitation assay lysis buffer was added. After complete cell lysis, the lysate was centrifuged at 10,000 g for 10 min at 4 °C to collect the supernatant. For sodium dodecyl sulfate-polyacrylamide gel electrophoresis, 12% separating gel and 5% stacking gel were prepared. Each lane was loaded with 40 μg of total protein. Electrophoresis was performed at a constant voltage of 80 V until the dye front reached the bottom of the stacking gel. The process was then continued at 120 V until the bromophenol blue reached the bottom of the gel. Transfer was performed at 4 °C using polyvinylidene difluoride (PVDF) membranes that had been pre-soaked in methanol for 2 min. The transfer apparatus was assembled according to standard protocols, and the transfer was carried out at a constant current of 400 mA. After transfer, the PVDF membrane was washed once with tris-buffered saline with Tween-20 (TBST) and then blocked with 5% bovine serum albumin buffer for 1 h at 37 °C, with shaking. The membrane was incubated with the primary antibody overnight. It was then incubated with horseradish peroxidase (HRP)-conjugated secondary antibody for 1 h at room temperature, with shaking. The membrane was washed with TBST, developed using enhanced chemiluminescence reagent, and imaged using an automated chemiluminescence analyzer. Incubation with the GAPDH internal reference antibody and the subsequent steps followed the same procedure. The antibodies used are listed in **Table 4**.

**Table 4:** Antibodies used for Western blot analysis.

Antibody	Dilution	Company	Catalog number
FEM1C	1:1000	San Ying	14037-1-AP
SUZ12	1:1000	Abcam	ab12073
GAPDH	1:10000	Proteintech	60004-1-Ig
-HRP-conjugated AffiniPure goat anti-rabbit IgG (H+L)	1:2000	Proteintech	SA00001-2
HRP-conjugated AffiniPure goat anti-mouse IgG (H+L)	1:2000	Proteintech	SA00001-1

Abcam, Cambridge, UK.

H+L, heavy and light chains; HRP, horseradish peroxidase; IgG, immunoglobulin G

### 2.13 m6A methylation quantification

The total RNA was extracted from the human peripheral blood samples using TRIzol LS Reagent according to the manufacturer’s instructions. Relative quantification of the m6A methylation was performed using the m6A RNA Methylation Quantification Kit (P-9005-96, Epigentek, Farmingdale, NY, USA). Absorbance was measured at 450 nm using a FlexStation 3 multi-mode microplate reader. The percentage of m6A in the total RNA was calculated using the formula provided by the kit manufacturer.

## 3 Results

### 3.1 Identification and GO analysis of ALS-associated m6A-SNPs

**Figure 1:**
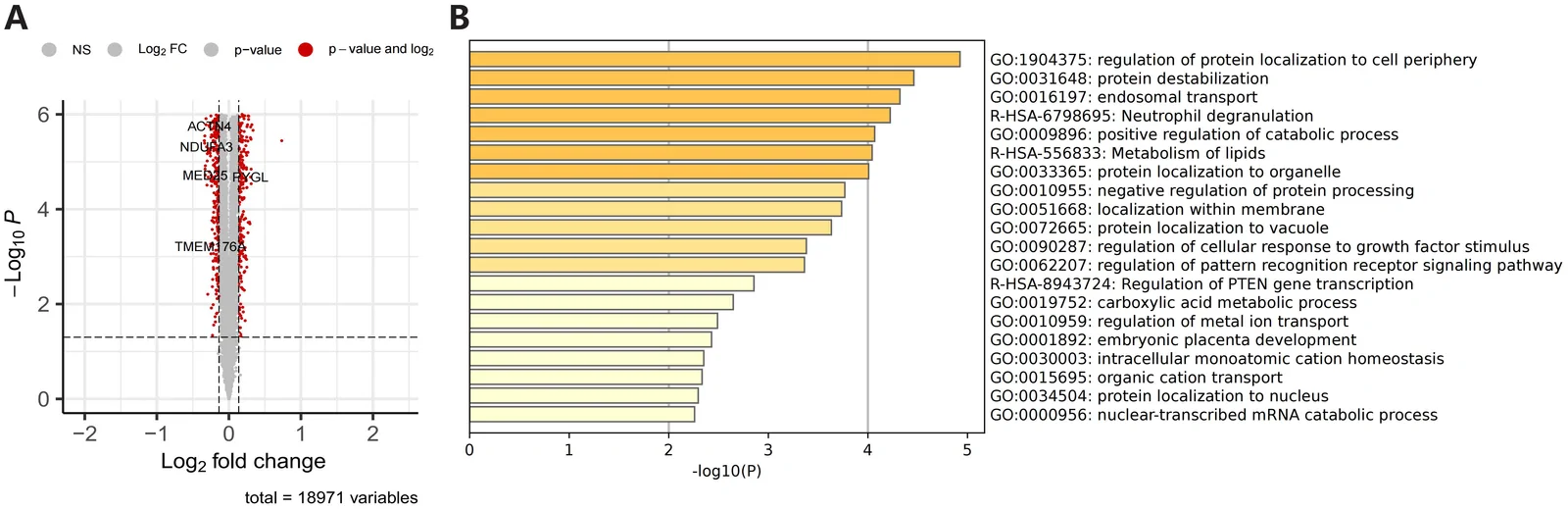
DEG and GO analyses of ALS-associated genes. **(A)** Analysis of DEGs in patients with amyotrophic lateral sclerosis; **(B)** GO enrichment analysis of the 109 genes associated with ALS that harbor m6A-SNPs with cis-eQTL. ALS, amyotrophic lateral sclerosis; cis-eQTL, cis-expression quantitative trait locus; DEGs, differentially expressed genes; GO, gene ontology; m6A-SNPs, N6-methyladenosine-associated single nucleotide polymorphisms.

To identify ALS-associated m6A-SNPs, we cross-referenced filtered SNPs and genes (threshold FDR <0.05) from the eQTL database with m6A-SNPs and genes from the RMVar database. This yielded potential m6A-SNPs with cis-eQTL signals. We then performed DEG analysis on the ALS bulk transcriptome data (GSE112676) using thresholds |log2FC| >0.25, padj <0.05. A total of 805 DEGs were obtained, comprising 519 upregulated and 286 downregulated genes (**Figure 1A**). Cross-checking the genes corresponding to m6A-SNPs with eQTL signals with these DEGs yielded 109 overlapping genes.

GO enrichment analysis describes the gene functions (BP, MF, CC) in biological systems. ALS-associated m6A-SNPs with BH-adjusted *p-*values <0.05 were considered significantly enriched. We used Metascape to perform GO enrichment analysis of the 109 intersecting genes (**Figure 1B**). The results showed significant enrichment in the regulation of protein localization to cell peripheries, protein destabilization, and endosomal transport.

### 3.2 Screening and validation of ALS diagnostic marker genes using machine learning

**Figure 2:**
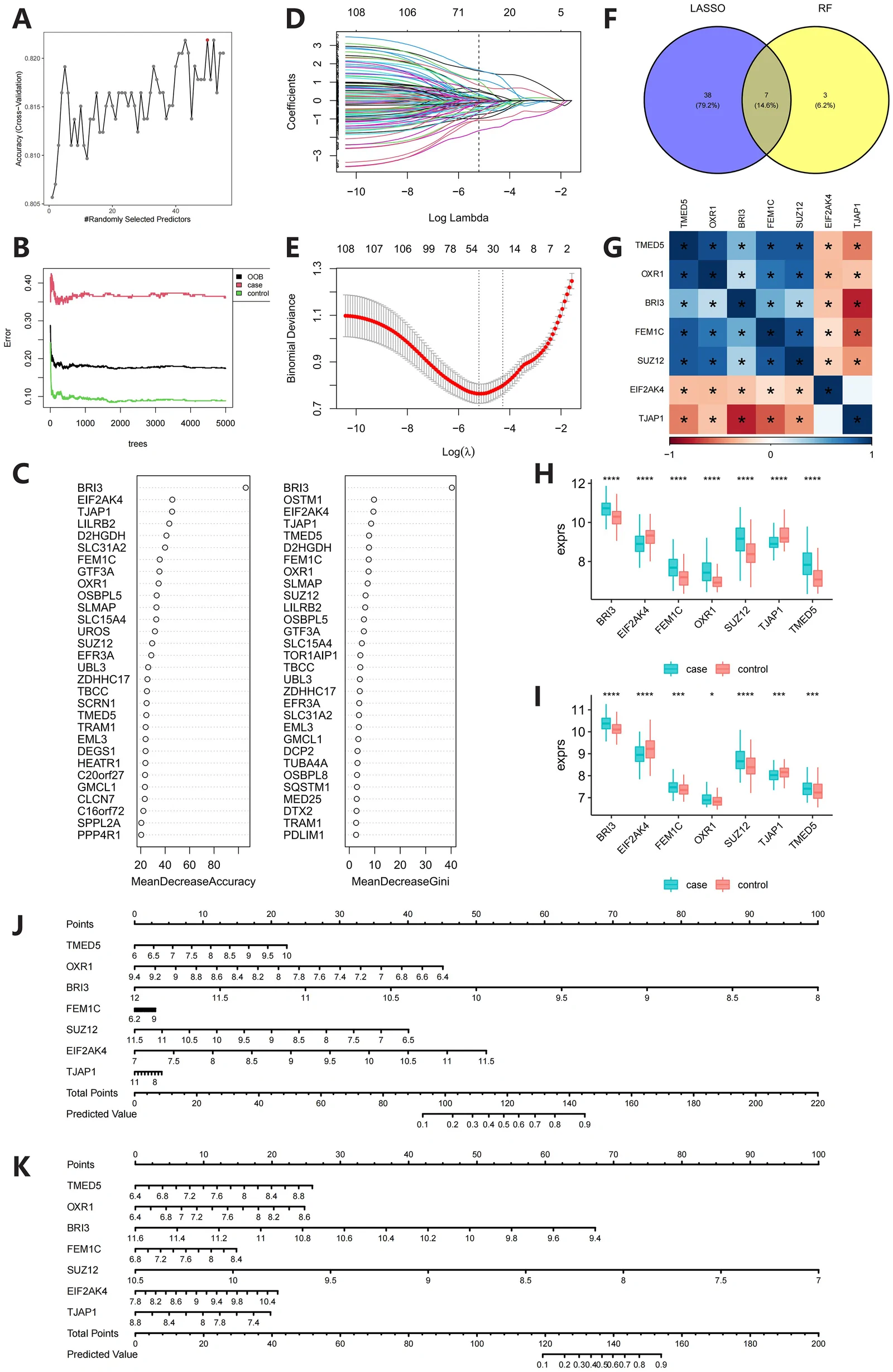
Identification and validation of diagnostic marker genes and model construction in ALS. **(A-C)** Screening of key genes using random forest: **(A)** tuning of the mtry parameter; **(B)** tuning of the ntree parameter; **(C)** ranking of gene importance based on MeanDecreaseGini. **(D-E)** LASSO feature selection: **(D)** coefficient paths; **(E)** cross-validation for selection of the optimal lambda (λ). **(F)** Venn diagram showing the intersection of random-forest and LASSO-selected genes; **(G)** correlation heatmap of the seven candidate markers; **(H-I)** expression levels in the training cohort (GSE112676) and independent validation cohort (GSE112680); **(J-K)** presentation and external evaluation of the same seven-gene model. The model was fitted in GSE112676, and its coefficients were applied unchanged to GSE112680 without model reconstruction or coefficient re-estimation. ALS, amyotrophic lateral sclerosis; LASSO, least absolute shrinkage and selection operator; MeanDecreaseGini, mean decrease in the Gini coefficient. *P < 0.05, ***P < 0.001, ****P < 0.0001.

Using ALS status as the endpoint, we performed random forest analysis on the 109 candidate genes associated with ALS-related m6A-SNPs. After fivefold cross-validation, the highest accuracy was achieved at mtry = 50 (**Figure 2A**), and the error rate stabilized at ntree = 5000 (**Figure 2B**). Based on MeanDecreaseGini (the mean decrease in the Gini coefficient) ranking, the top 10 genes were BRI3, OSTM1, EIF2AK4, TJAP1, TMED5, D2HGDH, FEM1C, OXR1, SLMAP, and SUZ12 (**Figure 2C**).

In our LASSO analysis, we performed cross-validation using “cv.glmnet” to select the optimal lambda parameter (**Figure 2D**). As the penalty increased, the coefficient paths of the variables decreased (**Figure 2E**). Variables with non-zero coefficients at the optimal lambda were considered important features. On this basis, we identified 45 important genes with non-zero coefficients.

In our random forest analysis, the top 10 genes ranked by MeanDecreaseGini were treated as important features. Intersecting these genes with the LASSO-selected set identified seven candidate markers: TMED5, OXR1, BRI3, FEM1C, SUZ12, EIF2AK4, and TJAP1 (**Figure 2F**). In 100 stratified bootstrap resampling iterations, each retained marker met the prespecified stability criterion of a selection frequency >=80%, supporting reproducibility of feature selection within the training data. Correlation analysis based on the expression levels of these markers revealed significant co-expression patterns (**Figure 2G**).

Using random forest and LASSO, we screened for the diagnostic marker genes in a training set (GSE112676). Validation in an independent dataset (GSE112680) confirmed that the seven diagnostic marker genes showed expression patterns consistent with those observed in the training set. Among these genes, TMED5, OXR1, BRI3, FEM1C, and SUZ12 showed increased expression in ALS samples, whereas EIF2AK4 and TJAP1 exhibited decreased expression compared with controls (**Figures 2H, I**). These results support the potential diagnostic value of the identified marker genes in ALS.

We next constructed a seven-gene model and a training-derived nomogram (**Figures 2J, K**). In the training cohort, the combined model achieved an AUC of 0.846 (95% CI 0.814-0.878), compared with lower AUCs for the individual markers. When the training-derived coefficients were applied unchanged to GSE112680, the model retained moderate discrimination, with an AUC of 0.770 (95% CI 0.717-0.823) (**Supplementary Table 2**; **Supplementary Figure 1**). However, external calibration was poor: the validation calibration intercept was 2.178, the slope was 1.117, the Brier score was 0.332, and the Hosmer-Lemeshow test was significant (p<0.001) (**Supplementary Table 3**). Thus, the external cohort supported retained discrimination but not well-calibrated absolute risk prediction.

### 3.3 Immune microenvironment analysis in healthy controls and ALS patients

To understand the immunopathological mechanisms underlying ALS, we used CIBERSORT to compare the immune cell composition of our samples from the ALS group and the control group.

**Figure 3:**
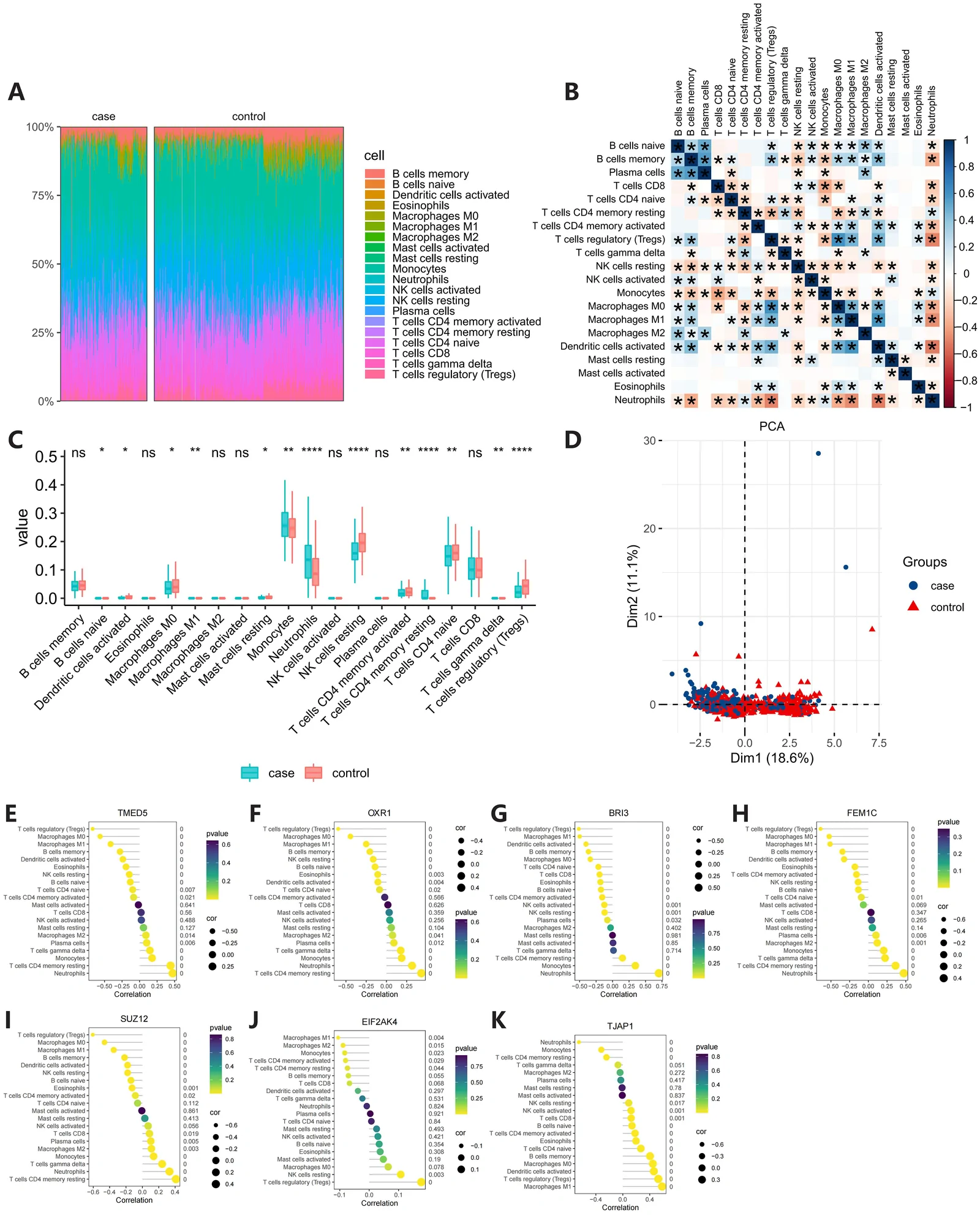
Immune cell infiltration and correlation with diagnostic marker genes in ALS. **(A)** Bar plot showing the relative proportions of 22 immune cell types in each sample (ALS vs. control); **(B)** Heatmap of correlations between the proportions of infiltration of different immune cells. Asterisks (*) indicate significant correlations (*p* < 0.05); **(C)** Boxplots showing differences in the infiltration levels of specific immune cell types between ALS patients and healthy controls; **(D)** PCA plot based on the immune cell infiltration profiles; **(E–K)** Heatmaps showing the Spearman’s correlation coefficients between each diagnostic gene and the infiltration levels of various immune cells. ALS, amyotrophic lateral sclerosis; PCA, principal component analysis.

**Figure 4:**
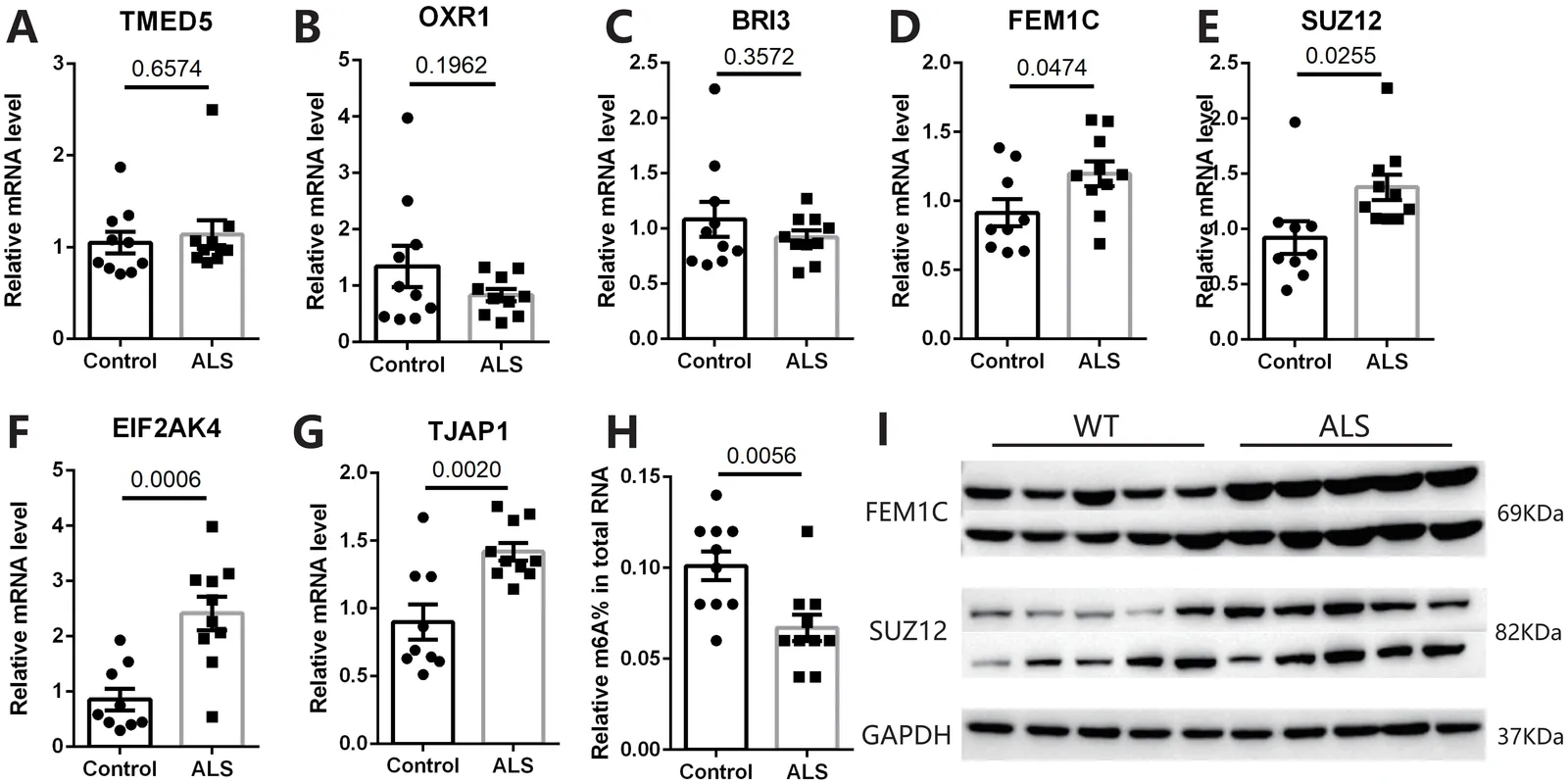
Validation of gene expression and global m6A levels in the peripheral blood of ALS patients. **(A–G)** Relative mRNA expression levels of **(A)** TMED5; **(B)** OXR1; **(C)** BRI3; **(D)** FEM1C; **(E)** SUZ12; **(F)** EIF2AK4; **(G)** TJAP1, as measured by qRT-PCR. **(H)** Global m6A modification levels in total RNA; **(I)** Western blot analysis of FEM1C and SUZ12 protein expression levels. Data are presented as mean ± SEM. Student’s t-tests were used for statistical analysis. ALS, amyotrophic lateral sclerosis; BRI3, brain protein I3; EIF2AK4, eukaryotic translation initiation factor 2 alpha kinase 4; FEM1C, fem-1 homolog C; m6A, N6-methyladenosine; mRNA, messenger RNA; OXR1, oxidation resistance 1; qRT-PCR, quantitative real-time polymerase chain reaction; RNA, ribonucleic acid; SEM, standard error of the mean; SUZ12, SUZ12 polycomb repressive complex 2 subunit; TJAP1, tight junction associated protein 1; TMED5, transmembrane emp24 domain-containing protein 5.

The CIBERSORT analysis estimated the relative proportions of 22 immune-cell types in each sample (**Figure 3A**). Correlation analysis revealed associations between multiple inferred immune-cell populations (**Figure 4B**). Boxplot comparisons showed differences in monocytes, neutrophils, and selected T-cell subsets, including naive CD4 T cells and memory CD8 T cells, between ALS and control samples (p<0.05) (**Figure 3C**). PCA showed partial separation but substantial overlap between groups (**Figure 3D**). Spearman correlation analyses further identified associations between marker-gene expression and inferred immune-cell proportions (**Figures 3E–K**). These findings indicate that marker expression covaries with inferred immune-cell composition; they do not establish that the marker genes regulate immune infiltration.

### 3.4 Consensus clustering analysis based on diagnostic marker genes

Different molecular subtypes of a disease may correspond to different pathological mechanisms and clinical features. Clustering analysis can identify any subtypes with potential differences in pathology, clinical presentation, and prognosis, aiding in patient stratification for targeted therapy and improving diagnostic accuracy.

Based on the identified diagnostic marker genes, we performed clustering analysis of our ALS patient sample using non-negative matrix factorization (NMF). The cophenetic correlation coefficient curve suggested an optimal cluster number of 2 (k = 2) (**Supplementary Figure 2**).

**Figure 5:**
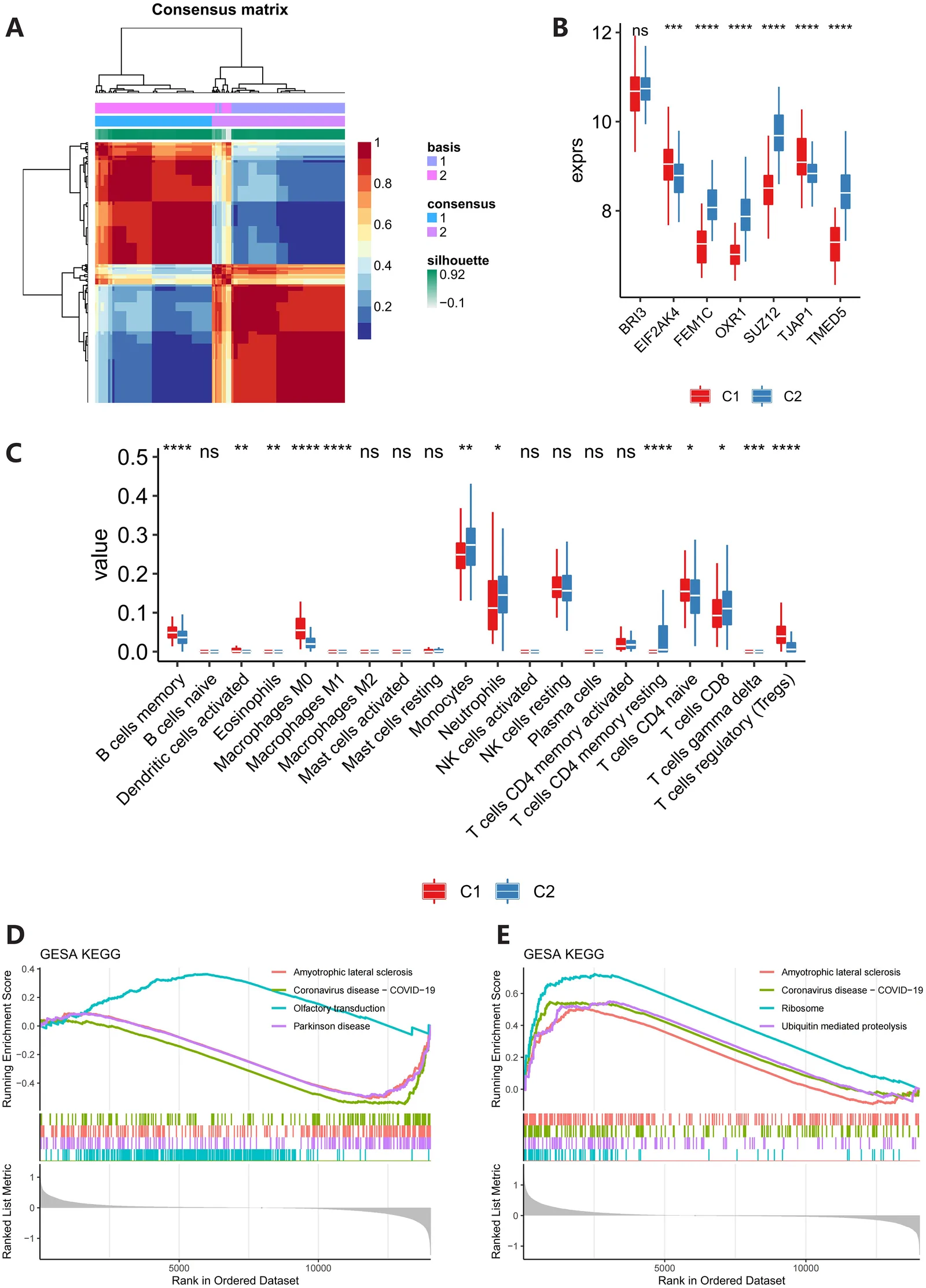
Molecular subtyping of ALS by NMF and characterization of immune and pathway features. **(A)** NMF clustering identified two ALS subtypes (C1 and C2); **(B)** Expression levels of diagnostic marker genes in NMF subtypes of ALS (C1 and C2); **(C)** Comparison of immune cell infiltration between NMF subtypes of ALS (C1 and C2); **(D, E)** GSEA for NMF subtypes of ALS. **(D)** Enrichment plot for the C1 subgroup; **(E)** Enrichment plot for the C2 subgroup. ALS, amyotrophic lateral sclerosis; GSEA, gene set enrichment analysis; NMF, non-negative matrix factorization. *P < 0.05, **P < 0.01, ***P < 0.001, ****P < 0.000.

The diagnostic marker genes effectively stratified the ALS patients into two distinct subtypes, which we designated C1 and C2 (**Figure 5A**). The expression of the diagnostic marker genes differed between these two (**Figure 5B**). In the C1 subtype, EIF2AK4 and TJAP1 were highly expressed, while FEM1C, OXR1, SUZ12, and TMED5 were highly expressed in the C2 subtype.

Comparing immune cell infiltration levels between the NMF subtypes also revealed differences in immune responses. Consistent with the overall immune analysis, we found significant differences in the infiltration of monocytes, T cells, and resting memory CD4 T cells (**Figure 5C**). We used gene set enrichment analysis (GSEA) to identify significantly enriched biological pathways in each subtype. We found significant enrichment of the ALS pathway in both subgroups (**Figures 5D, E**).

### 3.5 Integrative analysis of m6A modifications, m6A-SNP–RBP interactions, and diagnostic marker genes expression in ALS

To assess whether predicted m6A sites were located near the selected SNP loci, we compared the genomic positions of the RMVar-derived m6A-SNPs with SRAMP predictions. Predicted medium- or high-confidence m6A sites were located near rs1060622 (TMED5), rs28924709 (OXR1), rs6947208 (BRI3), rs73253005 (FEM1C), rs15654 (SUZ12), rs7173301 (EIF2AK4), and rs78262217 (TJAP1) (**Supplementary Figures 3A-G**). These results represent sequence-based predictions rather than direct measurement of site-specific m6A modification.

Using the UCSC Genome Browser (GRCh37/hg19), we examined the genomic context of the selected loci. The SNP regions overlapped or lay near annotations consistent with chromatin accessibility and RBP-binding regions (**Supplementary Figures 4A-G**). These observations are hypothesis-generating and suggest potential regulatory contexts, but they do not demonstrate that the variants alter m6A modification, RBP binding, RNA stability, or gene expression.

The expression levels of the seven diagnostic marker genes were further validated using peripheral blood samples from patients with ALS and controls. A qRT-PCR analysis revealed significantly upregulated relative mRNA expression levels of FEM1C, SUZ12, EIF2AK4, and TJAP1 in the ALS group (**Figures 4A–G**). Consistent with our mRNA findings, the expression levels of SUZ12 and FEM1C proteins were also significantly elevated in the ALS group (**Figure 4I**). Importantly, the global m6A modification level in total RNA from peripheral blood was significantly downregulated in the ALS group compared to the control group (**Figure 4H**).

## 4 Discussion

ALS remains a fatal neurodegenerative disease with substantial diagnostic and therapeutic challenges (1). In this study, we integrated eQTLGen, RMVar, and ALS whole-blood transcriptomic data to identify 109 candidate m6A-SNP-related genes. Machine-learning screening yielded seven candidate markers. The seven-gene model was developed in GSE112676 and evaluated in the independent GSE112680 cohort, whereas NMF-based clustering was used only as an exploratory analysis of molecular heterogeneity and was not independently validated.

m6A is a prevalent epitranscriptomic modification that contributes to regulation of RNA stability, splicing, and translation (23–26). m6A dysregulation has been reported in several neurodegenerative disorders, including AD and PD (26, 28, 29, 35), but disease specificity cannot be inferred from an ALS-versus-control design. Neurodegenerative diseases also differ substantially in their dominant pathological substrates, including amyloid-beta/tau pathology in AD, alpha-synuclein pathology in PD, and TDP-43 proteinopathy in most ALS and a subset of FTD cases (17, 36). Accordingly, direct comparative cohorts including ALS, AD, FTD, PD, and clinically relevant ALS mimics will be required before m6A-related signatures can be considered for differential diagnosis.

TDP-43 is an RNA-binding protein involved in RNA splicing, transport, stability, and translation, and abnormal TDP-43 redistribution is a central feature of ALS-FTD biology (17, 20, 37). m6A modification can influence RNA fate and interactions with RNA-binding proteins, and experimental work has linked RNA methylation to TDP-43 binding and toxicity (38). In the present study, the selected loci were found near predicted m6A sites and annotated RBP-binding regions, providing a plausible context for further mechanistic work. However, these in silico associations do not show that the specific m6A-SNPs alter TDP-43-related pathways, and this interpretation should therefore remain hypothesis-generating.

The 109 candidate genes were enriched in biological processes related to protein localization, destabilization, and endosomal transport. These pathways are compatible with broader disturbances of protein and cellular homeostasis in neurodegeneration (29, 39), but enrichment analysis alone cannot establish a causal role of m6A dysregulation in ALS.

The seven candidate markers map to several biologically relevant processes. OXR1 participates in oxidative-stress defense and has modified neurodegenerative phenotypes in experimental ALS models (40, 41). BRI3 is a brain-expressed membrane-associated protein and has appeared in ALS blood-expression analyses, although its direct ALS mechanism remains uncertain (42–44). EIF2AK4/GCN2 contributes to the integrated stress response and translational control under cellular stress (45–47). FEM1C participates in CRL2-mediated ubiquitin-dependent protein turnover (48, 49), whereas SUZ12 is a core component of PRC2 and contributes to chromatin-based transcriptional regulation (50, 51). TMED5 belongs to the p24/TMED family involved in secretory-pathway trafficking (52), and TJAP1 is a tight-junction-associated protein implicated in barrier and junctional regulation (53). These functions provide biological plausibility, but direct ALS-specific causal roles remain unproven for several markers.

In the exploratory clinical cohort, FEM1C and SUZ12 were increased at both mRNA and protein levels. This finding supports their candidacy for further study but does not establish a vital or causal role in ALS pathogenesis. The NMF analysis identified two expression-defined clusters, C1 and C2, with differences in inferred immune-cell composition. These clusters should be regarded as exploratory molecular groupings rather than validated clinical subtypes, and the present data do not support assigning either cluster to an earlier or later disease stage. The observed differences in T-cell, monocyte, and neutrophil-related signals are consistent with prior evidence of peripheral immune alterations in ALS (54–58).

The correlations between the seven markers and inferred immune-cell proportions are also associative. m6A-dependent regulation can influence immune-cell homeostasis in other biological contexts (59), providing a rationale for future mechanistic studies, but our CIBERSORT analysis cannot determine whether the candidate genes cause immune dysregulation or whether their expression instead reflects changes in blood-cell composition.

Global m6A modification in total peripheral-blood RNA was reduced in the ALS pilot cohort. A reduction in global m6A has also been reported in C9ORF72-ALS/FTD models and patient-derived material (60), while altered m6A regulation has been described in PD (61). Notably, FEM1C and SUZ12 expression increased despite the reduction in global m6A. The present data do not establish that reduced global m6A directly causes these gene-expression changes; site-specific methylation and functional experiments will be required to define any regulatory relationship.

From a biomarker perspective, the seven-gene model showed moderate external discrimination, but its external calibration was substantially impaired. Therefore, the current model should be considered a research classifier rather than a clinically deployable probability model. The discordant expression direction of EIF2AK4 and TJAP1 between the GEO cohorts and the qRT-PCR pilot cohort further argues for cautious interpretation. Differences in cohort composition, sample size, disease stage, population background, technical platform, and peripheral-blood cell composition could contribute to these discrepancies.

This study has several limitations. First, the transcriptomic cohorts primarily compared ALS with non-neurological controls; therefore, the analysis does not establish specificity against ALS mimics or other neurodegenerative diseases. Second, the external validation retained discrimination but showed substantial miscalibration, indicating that recalibration and further independent testing are necessary before clinical use. Third, age, treatment, disease duration at sampling, and measured blood-cell counts were incompletely available in the public cohorts, limiting covariate adjustment and interpretation of immune-infiltration analyses.Fourth, the clinical validation cohort included only 10 ALS patients and 10 controls and should be considered exploratory. The small sample size limits precision and generalizability, and the discordant qRT-PCR direction of EIF2AK4 and TJAP1 requires confirmation in larger, well-characterized cohorts. Fifth, the SRAMP and UCSC analyses are computational predictions and annotations rather than direct measurements of site-specific m6A modification or RBP binding. Finally, functional experiments such as site-specific m6A mapping, perturbation of the candidate variants or genes, and neuronal or disease-relevant cellular assays were not performed. Such studies will be required to determine whether the identified m6A-SNPs directly influence RNA modification, gene expression, TDP-43-related RNA biology, or ALS-relevant cellular phenotypes.

## 5 Conclusion

By integrating eQTL data, m6A-SNP annotations, and whole-blood transcriptomic profiles, this study identified seven candidate m6A-SNP-related markers associated with ALS. The combined model showed moderate external discrimination but substantial calibration error, indicating that recalibration and further independent validation are required before clinical use. Exploratory analyses identified differences in inferred immune-cell composition and two NMF-derived molecular clusters, while SRAMP and UCSC analyses identified predicted m6A sites and annotated RBP-binding regions near the selected variants. In a pilot clinical cohort, FEM1C and SUZ12 were increased at the mRNA and protein levels and global RNA m6A was reduced. These findings provide hypotheses for further biomarker and mechanistic studies rather than evidence for established early-diagnostic or subtype-stratification utility.

## Data Availability

The original contributions presented in the study are included in the article/**Supplementary Material**. Further inquiries can be directed to the corresponding authors.

## Supplementary material

Supplementary Figure 1 ROC analysis of identified ALS marker genes. **(A–G)** ROC curves for individual genes in the training set; **(H)** ROC curve for the nomogram model in the training set; **(I–O)** ROC curves for individual genes in the validation set; **(P)** ROC curve for the nomogram model in the validation set. ALS, amyotrophic lateral sclerosis; ROC, receiver operating characteristic

Supplementary Figure 2 NMF cluster analysis of patients with ALS based on diagnostic marker genes. Plot of cophenetic correlation coefficient versus cluster number (k). ALS, amyotrophic lateral sclerosis; NMF, non-negative matrix factorization

Supplementary Figure 3 SRAMP prediction of m6A modification sites near the identified m6A-SNPs in ALS. **(A)** rs1060622 (TMED5); **(B)** rs28924709 (OXR1); **(C)** rs6947208 (BRI3); **(D)** rs73253005 (FEM1C); **(E)** rs15654 (SUZ12); **(F)** rs7173301 (EIF2AK4); **(G)** rs78262217 (TJAP1). ALS, amyotrophic lateral sclerosis; BRI3, brain protein I3; EIF2AK4, eukaryotic translation initiation factor 2 alpha kinase 4; FEM1C, fem-1 homolog C; m6A, N6-methyladenosine; m6A-SNPs, N6-methyladenosine-associated single nucleotide polymorphisms; OXR1, oxidation resistance 1; RNA, ribonucleic acid; SRAMP, sequence-based RNA adenosine methylation site predictor; SUZ12, SUZ12 polycomb repressive complex 2 subunit; TJAP1, tight junction associated protein 1; TMED5, transmembrane emp24 domain-containing protein 5

Supplementary Figure 4 Genomic browser views (UCSC) of the m6A-SNPs loci in ALS. **(A)** rs1060622 (TMED5); **(B)** rs28924709 (OXR1); **(C)** rs6947208 (BRI3); **(D)** rs73253005 (FEM1C); **(E)** rs15654 (SUZ12); **(F)** rs7173301 (EIF2AK4); **(G)** rs78262217 (TJAP1). ALS, amyotrophic lateral sclerosis; BRI3, brain protein I3; EIF2AK4, eukaryotic translation initiation factor 2 alpha kinase 4; FEM1C, fem-1 homolog C; m6A-SNPs, N6-methyladenosine-associated single nucleotide polymorphisms; OXR1, oxidation resistance 1; SUZ12, SUZ12 polycomb repressive complex 2 subunit; TJAP1, tight junction associated protein 1; TMED5, transmembrane emp24 domain-containing protein 5; UCSC, University of California Santa Cruz database

## References

[1] Hardiman O Al-Chalabi A Chio A Corry EM Logroscino G Robberecht W . Amyotrophic lateral sclerosis. Nat Rev Dis Primers. (2017) 3:17071. doi: 10.1007/978-1-84996-011-3_7 28980624

[2] Brooks BR Jorgenson JA Newhouse BJ Shefner JM Agnese W . Edaravone in the treatment of amyotrophic lateral sclerosis: efficacy and access to therapy - a roundtable discussion. Am J Manag Care. (2018) 24:S175–86. 29693363

[3] Debove C Zeisser P Salzman PM Powe LK Truffinet P . The Rilutek (riluzole) Global Early Access Programme: an open-label safety evaluation in the treatment of amyotrophic lateral sclerosis. Amyotroph Lateral Scler Other Motor Neuron Disord. (2001) 2:153–8. doi: 10.1080/146608201753275508 11771772

[4] Ferrari R Kapogiannis D Huey ED Momeni P . FTD and ALS: a tale of two diseases. Curr Alzheimer Res. (2011) 8:273–94. doi: 10.2174/156720511795563700 21222600 PMC3801195

[5] Bennett SA Tanaz R Cobos SN Torrente MP . Epigenetics in amyotrophic lateral sclerosis: a role for histone post-translational modifications in neurodegenerative disease. Transl Res. (2019) 204:19–30. doi: 10.1016/j.trsl.2018.10.002 30391475 PMC6331271

[6] Al-Chalabi A Hardiman O . The epidemiology of ALS: a conspiracy of genes, environment and time. Nat Rev Neurol. (2013) 9:617–28. doi: 10.1038/nrneurol.2013.203 24126629

[7] Turner MR Hardiman O Benatar M Brooks BR Chio A de Carvalho M . Controversies and priorities in amyotrophic lateral sclerosis. Lancet Neurol. (2013) 12:310–22. doi: 10.1016/s1474-4422(13)70036-x 23415570 PMC4565161

[8] Chia R Chiò A Traynor BJ . Novel genes associated with amyotrophic lateral sclerosis: diagnostic and clinical implications. Lancet Neurol. (2018) 17:94–102. doi: 10.1016/s1474-4422(17)30401-5 29154141 PMC5901717

[9] DeJesus-Hernandez M Mackenzie IR Boeve BF Boxer AL Baker M Rutherford NJ . Expanded GGGGCC hexanucleotide repeat in noncoding region of C9ORF72 causes chromosome 9p-linked FTD and ALS. Neuron. (2011) 72:245–56. doi: 10.1016/j.neuron.2011.09.011 21944778 PMC3202986

[10] Rosen DR Siddique T Patterson D Figlewicz DA Sapp P Hentati A . Mutations in Cu/Zn superoxide dismutase gene are associated with familial amyotrophic lateral sclerosis. Nature. (1993) 362:59–62. doi: 10.1038/364362c0 8446170

[11] Sreedharan J Blair IP Tripathi VB Hu X Vance C Rogelj B . TDP-43 mutations in familial and sporadic amyotrophic lateral sclerosis. Science. (2008) 319:1668–72. doi: 10.1126/science.1154584 18309045 PMC7116650

[12] Kwiatkowski TJ Bosco DA LeClerc AL Tamrazian E Vanderburg CR Russ C . Mutations in the FUS/TLS gene on chromosome 16 cause familial amyotrophic lateral sclerosis. Science. (2009) 323:1205–8. doi: 10.1126/science.1166066 19251627

[13] Cirulli ET Lasseigne BN Petrovski S Sapp PC Dion PA Leblond CS . Exome sequencing in amyotrophic lateral sclerosis identifies risk genes and pathways. Science. (2015) 347:1436–41. doi: 10.1126/science.aaa3650 25700176 PMC4437632

[14] Taylor JP Brown RH Jr. Cleveland DW . Decoding ALS: from genes to mechanism. Nature. (2016) 539:197–206. doi: 10.1038/nature20413 27830784 PMC5585017

[15] Strong MJ Abrahams S Goldstein LH Woolley S Mclaughlin P Snowden J . Amyotrophic lateral sclerosis - frontotemporal spectrum disorder (ALS-FTSD): Revised diagnostic criteria. Amyotroph Lateral Scler Frontotemporal Degener. (2017) 18:153–74. doi: 10.1080/21678421.2016.1267768 28054827 PMC7409990

[16] Phukan J Pender NP Hardiman O . Cognitive impairment in amyotrophic lateral sclerosis. Lancet Neurol. (2007) 6:994–1003. doi: 10.1016/s1474-4422(07)70265-x 17945153

[17] Burrell JR Halliday GM Kril JJ Ittner LM Götz J Kiernan MC . The frontotemporal dementia-motor neuron disease continuum. Lancet. (2016) 388:919–31. doi: 10.1016/s0140-6736(16)00737-6 26987909

[18] Neumann M Sampathu DM Kwong LK Truax AC Micsenyi MC Chou TT . Ubiquitinated TDP-43 in frontotemporal lobar degeneration and amyotrophic lateral sclerosis. Science. (2006) 314:130–3. doi: 10.1126/science.1134108 17023659

[19] Geser F Martinez-Lage M Kwong LK Lee VMY Trojanowski JQ . Amyotrophic lateral sclerosis, frontotemporal dementia and beyond: the TDP-43 diseases. J Neurol. (2009) 256:1205–14. doi: 10.1007/s00415-009-5069-7 19271105 PMC2790321

[20] Ling SC Polymenidou M Cleveland DW . Converging mechanisms in ALS and FTD: disrupted RNA and protein homeostasis. Neuron. (2013) 79:416–38. doi: 10.1016/j.neuron.2013.07.033 23931993 PMC4411085

[21] Renton AE Majounie E Waite A Simón-Sánchez J Rollinson S Gibbs JR . A hexanucleotide repeat expansion in C9ORF72 is the cause of chromosome 9p21-linked ALS-FTD. Neuron. (2011) 72:257–68. doi: 10.1016/j.neuron.2011.09.010 21944779 PMC3200438

[22] Bennett SA Tanaz R Cobos SN Torrente MP . Epigenetics in amyotrophic lateral sclerosis: a role for histone post-translational modifications in neurodegenerative disease. Transl Res. (2019) 204:19–30. doi: 10.1016/j.trsl.2018.10.002 30391475 PMC6331271

[23] Li J Yang X Qi Z Sang Y Liu Y Xu B . The role of mRNA m(6)A methylation in the nervous system. Cell Biosci. (2019) 9:66. doi: 10.1186/s13578-019-0330-y 31452869 PMC6701067

[24] Fu Y Dominissini D Rechavi G He C . Gene expression regulation mediated through reversible m^6^A RNA methylation. Nat Rev Genet. (2014) 15:293–306. doi: 10.1038/nrg3724 24662220

[25] Coots RA Liu XM Mao Y Dong L Zhou J Wan J . m(6)A Facilitates eIF4F-Independent mRNA Translation. Mol Cell. (2017) 68:504–14. doi: 10.1016/j.molcel.2017.10.002 29107534 PMC5913006

[26] Deng J Chen X Chen A Zheng X . m(6)A RNA methylation in brain injury and neurodegenerative disease. Front Neurol. (2022) 13:995747. doi: 10.3389/fneur.2022.995747 36158961 PMC9493472

[27] Guo F Kang J Xu J Wei S Tao J Dong Y . Genome-wide identification of m(6)A-associated single nucleotide polymorphisms in complex diseases of nervous system. Neurosci Lett. (2023) 817:137513. doi: 10.1016/j.neulet.2023.137513 37827449

[28] Qiu X He H Huang Y Wang J Xiao Y . Genome-wide identification of m(6)A-associated single-nucleotide polymorphisms in Parkinson's disease. Neurosci Lett. (2020) 737:135315. doi: 10.1016/j.neulet.2020.135315 32827573

[29] Jan SM Fahira A Shi Y Khan MI Jamal A Mahmood A . Integrative genomic analysis of m6a-SNPs identifies potential functional variants associated with alzheimer's disease. ACS Omega. (2023) 8:13332–41. doi: 10.1021/acsomega.3c00696 37065064 PMC10099436

[30] Brooks BR Miller RG Swash M Munsat TL . El Escorial revisited: revised criteria for the diagnosis of amyotrophic lateral sclerosis. Amyotroph Lateral Scler Other Motor Neuron Disord. (2000) 1:293–9. doi: 10.1080/146608200300079536 11464847

[31] Võsa U Claringbould A Westra HJ Bonder MJ Deelen P Zeng B . Large-scale cis- and trans-eQTL analyses identify thousands of genetic loci and polygenic scores that regulate blood gene expression. Nat Genet. (2021) 53:1300–10. doi: 10.1038/s41588-021-00924-w PMC8432599 34475573

[32] Luo X Li H Liang J Zhao Q Xie Y Ren J . RMVar: an updated database of functional variants involved in RNA modifications. Nucleic Acids Res. (2021) 49:D1405–12. doi: 10.1093/nar/gkaa811 33021671 PMC7779057

[33] Zhou Y Zeng P Li YH Zhang Z Cui Q . SRAMP: prediction of mammalian N6-methyladenosine (m6A) sites based on sequence-derived features. Nucleic Acids Res. (2016) 44:e91. doi: 10.1093/nar/gkw104 26896799 PMC4889921

[34] Kent WJ Sugnet CW Furey TS Roskin KM Pringle TH Zahler AM . The human genome browser at UCSC. Genome Res. (2002) 12:996–1006. doi: 10.1101/gr.229102 12045153 PMC186604

[35] Han M Liu Z Xu Y Liu X Wang D Li F . Abnormality of m6A mRNA Methylation Is Involved in Alzheimer's Disease. Front Neurosci. (2020) 14:98. doi: 10.3389/fnins.2020.00098 32184705 PMC7058666

[36] Dugger BN Dickson DW . Pathology of neurodegenerative diseases. Cold Spring Harb Perspect Biol. (2017) 9. doi: 10.1101/cshperspect.a028035 28062563 PMC5495060

[37] Mann JR Gleixner AM Mauna JC Gomes E DeChellis-Marks MR Needham PG . RNA binding antagonizes neurotoxic phase transitions of TDP-43. Neuron. (2019) 102:321–38. doi: 10.1016/j.neuron.2019.01.048 30826182 PMC6472983

[38] McMillan M Gomez N Hsieh C Bekier M Li X Miguez R . RNA methylation influences TDP43 binding and disease pathogenesis in models of amyotrophic lateral sclerosis and frontotemporal dementia. Mol Cell. (2023) 83:219–36. doi: 10.1016/j.molcel.2022.12.019 36634675 PMC9899051

[39] Hetz C Saxena S . ER stress and the unfolded protein response in neurodegeneration. Nat Rev Neurol. (2017) 13:477–91. doi: 10.1038/nrneurol.2017.99 28731040

[40] Liu KX Edwards B Lee S Finelli MJ Davies B Davies KE . Neuron-specific antioxidant OXR1 extends survival of a mouse model of amyotrophic lateral sclerosis. Brain. (2015) 138:1167–81. doi: 10.1093/brain/awv039 25753484 PMC4407188

[41] Oliver PL Finelli MJ Edwards B Bitoun E Butts DL Becker EBE . Oxr1 is essential for protection against oxidative stress-induced neurodegeneration. PloS Genet. (2011) 7:e1002338. doi: 10.1371/journal.pgen.1002338 22028674 PMC3197693

[42] Swindell WR Kruse CPS List EO Berryman DE Kopchick JJ . ALS blood expression profiling identifies new biomarkers, patient subgroups, and evidence for neutrophilia and hypoxia. J Transl Med. (2019) 17:170. doi: 10.1186/s12967-019-1909-0 31118040 PMC6530130

[43] Vidal R Calero M Révész T Plant G Ghiso J Frangione B . Sequence, genomic structure and tissue expression of Human BRI3, a member of the BRI gene family. Gene. (2001) 266:95–102. doi: 10.1016/s0378-1119(01)00374-2 11290423

[44] Yang L Xiong Y Hu XF Du YH . MicroRNA-323 regulates ischemia/reperfusion injury-induced neuronal cell death by targeting BRI3. Int J Clin Exp Pathol. (2015) 8:10725–33. PMC4637598 26617783

[45] Bond S Lopez-Lloreda C Gannon PJ Akay-Espinoza C Jordan-Sciutto KL . The integrated stress response and phosphorylated eukaryotic initiation factor 2α in neurodegeneration. J Neuropathol Exp Neurol. (2020) 79:123–43. doi: 10.1093/jnen/nlz129 31913484 PMC6970450

[46] Pakos-Zebrucka K Koryga I Mnich K Ljujic M Samali A Gorman AM . The integrated stress response. EMBO Rep. (2016) 17:1374–95. doi: 10.15252/embr.201642195 27629041 PMC5048378

[47] Wek RC Jiang HY Anthony TG . Coping with stress: eIF2 kinases and translational control. Biochem Soc Trans. (2006) 34:7–11. doi: 10.1042/bst0340007 16246168

[48] Dubey AA Krygier M Szulc NA Rutkowska K Kosińska J Pollak A . A novel de novo FEM1C variant is linked to neurodevelopmental disorder with absent speech, pyramidal signs and limb ataxia. Hum Mol Genet. (2023) 32:1152–61. doi: 10.1093/hmg/ddac276 36336956 PMC10026218

[49] Yan X Wang X Li Y Zhou M Li Y Song L . Molecular basis for ubiquitin ligase CRL2(FEM1C)-mediated recognition of C-degron. Nat Chem Biol. (2021) 17:263–71. doi: 10.1038/s41589-020-00703-4 33398170

[50] Margueron R Reinberg D . The Polycomb complex PRC2 and its mark in life. Nature. (2011) 469:343–9. doi: 10.1038/nature09784 21248841 PMC3760771

[51] Di Croce L Helin K . Transcriptional regulation by Polycomb group proteins. Nat Struct Mol Biol. (2013) 20:1147–55. doi: 10.1038/nsmb.2669 24096405

[52] Pastor-Cantizano N Montesinos JC Bernat-Silvestre C Marcote MJ Aniento F . p24 family proteins: Key players in the regulation of trafficking along the secretory pathway. Protoplasma. (2016) 253:967–85. doi: 10.1007/s00709-015-0858-6 26224213

[53] Burek M König A Lang M Fiedler J Oerter S Roewer N . Hypoxia-induced microRNA-212/132 alter blood-brain barrier integrity through inhibition of tight junction-associated proteins in human and mouse brain microvascular endothelial cells. Transl Stroke Res. (2019) 10:672–83. doi: 10.1007/s12975-018-0683-2 30617994 PMC6842347

[54] Sheean RK McKay FC Cretney E Bye CR Perera ND Tomas D . Association of regulatory T-cell expansion with progression of amyotrophic lateral sclerosis: A study of humans and a transgenic mouse model. JAMA Neurol. (2018) 75:681–9. doi: 10.1001/jamaneurol.2018.0035 29507931 PMC5885208

[55] Beers DR Henkel JS Zhao W Wang J Appel SH . CD4+ T cells support glial neuroprotection, slow disease progression, and modify glial morphology in an animal model of inherited ALS. Proc Natl Acad Sci USA. (2008) 105:15558–63. doi: 10.1073/pnas.0807419105 18809917 PMC2547419

[56] Murdock BJ Bender DE Kashlan SR Figueroa-Romero C Backus C Callaghan BC . Increased ratio of circulating neutrophils to monocytes in amyotrophic lateral sclerosis. Neurol Neuroimmunol Neuroinflamm. (2016) 3:e242. doi: 10.1212/nxi.0000000000000242 27308304 PMC4897983

[57] McCombe PA Lee JD Woodruff TM Henderson RD . The peripheral immune system and amyotrophic lateral sclerosis. Front Neurol. (2020) 11:279. doi: 10.3389/fneur.2020.00279 32373052 PMC7186478

[58] Beers DR Appel SH . Immune dysregulation in amyotrophic lateral sclerosis: Mechanisms and emerging therapies. Lancet Neurol. (2019) 18:211–20. doi: 10.1016/s1474-4422(18)30394-6 30663610

[59] Li HB Tong J Zhu S Batista PJ Duffy EE Zhao J . m6A mRNA methylation controls T cell homeostasis by targeting the IL-7/STAT5/SOCS pathways. Nature. (2017) 548:338–42. doi: 10.1038/nature23450 28792938 PMC5729908

[60] Li Y Dou X Liu J Xiao Y Zhang Z Hayes L . Globally reduced N6-methyladenosine (m6A) in C9ORF72-ALS/FTD dysregulates RNA metabolism and contributes to neurodegeneration. Nat Neurosci. (2023) 26:1328–38. doi: 10.1038/s41593-023-01374-9 37365312 PMC11361766

[61] He H Zhang Q Liao J Lei J Luo M Huang J . METTL14 is decreased and regulates m6A modification of α-synuclein in Parkinson's disease. J Neurochem. (2023) 166:609–22. doi: 10.1111/jnc.15882 37309980

