# Supplementary material for: Integrated multi-omics identification of m6A-SNP-related diagnostic biomarkers in amyotrophic lateral sclerosis
Source: Front Immunol. 2026 Aug 27;17:1914869. doi: 10.3389/fimmu.2026.1914869 (PMC13557987; doi:10.3389/fimmu.2026.1914869)
Supplement: Supplementary file 1 [file Table1.docx]

**Supplementary Table 1a.** **Cohort characteristics**

| **Characteristic** | **GSE112676_ALS** | **GSE112676_Control** | **GSE112680_ALS** | **GSE112680_Control** |
| --- | --- | --- | --- | --- |
| No. of subjects analysed | 233 | 508 | 164 | 137 |
| Sex, male | 143 (61.4%) | 278 (54.7%) | 96 (58.5%) | 79 (57.7%) |
| Sex, female | 90 (38.6%) | 230 (45.3%) | 68 (41.5%) | 58 (42.3%) |
| Age at symptom onset, years (mean ± SD [range]) | 62.4 ± 11.8 [24.2-88.5] | Not available | 61.9 ± 12.1 [23.1-86.4] | Not available |
| Site of onset, spinal | 143 (61.4%) | Not applicable | 108 (65.9%) | Not applicable |
| Site of onset, bulbar | 90 (38.6%) | Not applicable | 56 (34.1%) | Not applicable |
| Follow-up from onset, years (mean ± SD [range]) | 3.0 ± 2.1 [0.3-16.5] | Not applicable | 2.7 ± 1.7 [0.4-13.6] | Not applicable |

**Supplementary Table 1b.** **Data sources and preprocessing**

| **Item** | **GSE112676** | **GSE112680** |
| --- | --- | --- |
| GEO series | GSE112676 | GSE112680 |
| Platform | GPL6947 | GPL10558 |
| Array | Illumina HumanHT-12 v3 BeadChip | Illumina HumanHT-12 v4 BeadChip |
| Tissue | Whole blood | Whole blood |
| RNA source | PAXgene stabilised venous blood | PAXgene stabilised venous blood |
| Globin reduction | Not performed | Not performed |
| Samples in GEO series | 741 | 376 |
| ALS | 233 | 164 |
| Non-neurological controls | 508 | 137 |
| ALS-mimic disorders | 0 | 75 |
| ALS-mimics handling | None present in this series | Excluded before analysis |
| Samples analysed here | 741 | 301 |
| Normalisation | Quantile normalisation, log2 transformation (IlluminaGUI) | Quantile normalisation, log2 transformation (IlluminaGUI) |
| Quality control | Sex verified against Y-chromosome probes (JARID1D, RPS4Y1); PCA outliers removed by the depositors | Sex verified against Y-chromosome probes (JARID1D, RPS4Y1); PCA outliers removed by the depositors |
| Batch handling | Samples randomised across chips before hybridisation; surrogate variable analysis applied by the depositors across both series | Samples randomised across chips before hybridisation; surrogate variable analysis applied by the depositors across both series |
| Role in this study | Training cohort | Independent validation cohort |
| Censoring indicator | Deposited for patients; the GEO record does not document the coding direction, so it is not summarised here | Deposited for patients; the GEO record does not document the coding direction, so it is not summarised here |

**Supplementary Table 2.** **Diagnostic performance of individual markers and the seven-gene model based on ROC analysis**

| **Marker** | **Direction** | **Training_AUC_95CI** | **Validation_AUC_95CI** |
| --- | --- | --- | --- |
| BRI3 | Higher in ALS | 0.761 (0.722-0.800) | 0.712 (0.654-0.771) |
| EIF2AK4 | Lower in ALS | 0.297 (0.256-0.337) | 0.359 (0.296-0.422) |
| FEM1C | Higher in ALS | 0.732 (0.691-0.774) | 0.614 (0.551-0.678) |
| OXR1 | Higher in ALS | 0.748 (0.709-0.787) | 0.584 (0.520-0.648) |
| SUZ12 | Higher in ALS | 0.740 (0.700-0.779) | 0.640 (0.578-0.702) |
| TJAP1 | Lower in ALS | 0.293 (0.251-0.336) | 0.386 (0.323-0.450) |
| TMED5 | Higher in ALS | 0.758 (0.719-0.797) | 0.612 (0.547-0.676) |
| Seven-gene model | - | 0.846 (0.814-0.878) | 0.770 (0.717-0.823) |

**Supplementary Table 3.** **Calibration performance of the seven-gene diagnostic model**

| **Setting** | **mean_predicted** | **observed** | **intercept** | **slope** | **brier** | **hl_stat** | **hl_p** |
| --- | --- | --- | --- | --- | --- | --- | --- |
| Training cohort, apparent | 0.3144 | 0.3144 | 0 | 1 | 0.1397 | 17.9898 | 0.0213 |
| Validation, coefficients unchanged | 0.1963 | 0.5449 | 2.1781 | 1.1165 | 0.3321 | 330.3694 | 0 |
| Validation, intercept recalibrated | 0.5449 | 0.5449 | -0.0134 | 1.1165 | 0.1938 | 9.0843 | 0.3352 |
| Validation, within-cohort standardised | 0.2946 | 0.5449 | 1.1973 | 0.807 | 0.2689 | 164.8791 | 0 |

**Supplementary Table 4. Functional annotation of m6A-SNP-related diagnostic marker genes and their potential relevance to ALS pathology**

| Gene | Biological function | Potential relevance to ALS pathology |
| --- | --- | --- |
| TMED5 | TMED5 is a member of the p24/TMED family, whose proteins participate in ER-to-Golgi trafficking and organization of the secretory pathway [62]. | Because secretory-pathway and protein-homeostasis disturbances are relevant to neurodegeneration, altered TMED5 expression may reflect perturbation of these processes; an ALS-specific causal role has not been established [40,62]. |
| OXR1 | OXR1 participates in cellular defense against oxidative stress and protects against oxidative-stress-induced neurodegeneration [48]. | OXR1 has modified disease-related neurodegeneration in an experimental ALS model, supporting biological plausibility; the direction and clinical significance of peripheral-blood OXR1 expression remain to be clarified [41,48]. |
| BRI3 | BRI3 is a brain-expressed membrane-associated protein whose genomic structure and tissue expression have been characterized [45]. | BRI3 has appeared in ALS blood-expression biomarker analyses, but its direct mechanistic role in ALS remains uncertain [42,49]. |
| FEM1C | FEM1C is a substrate receptor of CRL2 ubiquitin ligase complexes involved in C-degron recognition and protein turnover [46]. | Given the importance of proteostasis in neurodegeneration, altered FEM1C expression may be associated with ubiquitin-dependent protein turnover; ALS-specific causality has not been demonstrated [40,44,46]. |
| SUZ12 | SUZ12 is a core component of Polycomb Repressive Complex 2 (PRC2) and contributes to chromatin regulation and transcriptional repression [50,51]. | Epigenetic dysregulation is relevant to neurodegeneration, but the direct contribution of altered SUZ12 expression to ALS remains hypothesis-generating [50,51]. |
| EIF2AK4 | EIF2AK4 (GCN2) is an eIF2α kinase that contributes to the integrated stress response and translational control under cellular stress [47,52]. | Dysregulated integrated-stress-response signaling can affect neuronal adaptation and survival; whether peripheral EIF2AK4 expression contributes directly to ALS is unknown [43,47,52]. |
| TJAP1 | TJAP1 is a tight-junction-associated protein implicated in endothelial barrier and tight-junction regulation [53]. | Its ALS-specific function remains largely unknown; the observed expression signal should therefore be interpreted as a candidate biomarker requiring functional validation [53]. |
